# On the Practical Teaching of Sanitary Science in National Schools

**Published:** 1855-06

**Authors:** W. H. Lyttelton


### ON THE PRACTICAL TEACHING OF SANITARY SCIENCE IN NATIONAL SCHOOLS.

By the Hon. and Rev. W. H. Lyttelton.

I wish to draw the attention of sanitary reformers to the importance of making the first principles of sanitary science a part of the regular course of instruction in all national and day schools.

It is evident that, until we are able to create a strong and intelligent public opinion among the working classes themselves in favour of sanitary reform, all interference of boards of guardians, or of other official persons, can be only partially and imperfectly successful.

Permanent sanitary officers invested with legal authority, such as are to be conferred upon us under Sir B. Hall's bill, will, indeed, be a great boon, and may do much. But so long as the poor are *unwilling* to obey sanitary laws, we shall be but like Sisyphus constantly rolling stones to the tops of hills, which will immediately roll down again. Every guardian of the poor will tell us how many unwearied Penelopes there are in every village and town, who, chivalrous in their resistance to authority, and love of dirt, will be always unweaving, under cover of darkness, the veil of decency and cleanliness which they were compelled by terrible sanitary suitors to weave in the daytime. "You can lead unwilling horses to the water, but you cannot make them drink" or wash themselves.

There is, therefore, but one remedy within our reach which will at all cover the whole extent of the evil; but there is one, namely, the creation—by means of education—of such an enlightened public opinion on these subjects as will constitute every intelligent and well disposed man a vigilant and interested inspector of nuisances in his own home and neighbourhood, ready and anxious to call in, if necessary, the arm of the law, to punish offenders, and enforce obedience; and we must also so instruct the minds of all in the great principles of health, that all stern messengers of the Divine mercy—such as the cholera—shall speak to them the message, which they are intended to speak to all, but which never reaches the minds of many on account of the thick veil of ignorance and prejudice with which they are surrounded.

Let, then, the principles of sanitary science, the practical consequences of good and evil which flow from obeying or neglecting its laws, be thoroughly taught in all schools. Let the clergyman and schoolmaster lose no opportunity of instilling right feelings and sound practical knowledge on this subject into the minds of the children. Let books upon it be read and made the subject of conversation with the girls by the schoolmistress, while they are at their work in the afternoons, or at other times.*

The advantage of teaching this science to children is by no means limited to its *direct* effects; it is an excellent means of mental education, and of leading them to realise the close connexion between scientific knowledge and practical life.

If we were content to aim only at being slave-drivers, forcing men to right conduct against their will, so long as we are present to drive them to it, we may look, in sanitary reform, as in other matter, to prisons, police courts, and public officers as our chief instruments. But if we aim higher and wish to make men *willing* "bondmen of duty," serving freely "in the light of truth" in our absence as well as in our presence, we must look in this, as in everything, to schools, as the great fountain head whence all good is to flow. In the work of sanitary reform, as well as in all other reform, if we wish to lay the foundations of our work deep, we must look in one direction above all—that is, to education.

*Hagley Rectory, June,* 1855.

* There is an excellent little *Manual of Public Health and Domestic Economy*, published by John Churchill for the "Metropolitan Working Classes' Association," which, besides being very well written, has the recommendation of being published by members of the working classes. I have long made use of this as a text book in our village school. But we very much want some lively and plainly written treatises (perhaps they would be most effective in the form of *conversations*) on sanitary science, for use in schools.

